# The association between novel urinary kidney damage biomarkers and coronary atherosclerosis in an apparently healthy population

**DOI:** 10.1038/s41598-024-80321-5

**Published:** 2024-11-25

**Authors:** Yi-Ting Lin, Jonas Wuopio, Anders Larsson, Andrei Malinovschi, Tobias Feldreich, Gunnar Engström, Tove Fall, Johan Ärnlöv

**Affiliations:** 1 Division of Family Medicine, Department of Neurobiology, Care Sciences and Society, Karolinska Institutet, Huddinge, Sweden; 2 Department of Family Medicine, Kaohsiung Medical University Hospital, Kaohsiung Medical University, Kaohsiung, Taiwan; 3 Center for Clinical Research Dalarna, Uppsala University, Falun, Sweden; 4 Department of Medical Sciences, Clinical Chemistry, Uppsala University, Uppsala, Sweden; 5 Department of Medical Sciences, Clinical Physiology, Uppsala University, Uppsala, Sweden; 6 School of Health and Welfare, Dalarna University, Falun, Sweden; 7 Department of Clinical Sciences, Cardiovascular Epidemiology, Lund University, Malmö, Sweden; 8 Molecular Epidemiology and Science for Life Laboratory, Department of Medical Sciences, Uppsala University, Uppsala, Sweden

**Keywords:** Coronary atherosclerosis, Dickkopf-3, Epidermal growth factor, Kidney injury molecule-1, Osteopontin, Biomarkers, Diseases, Medical research, Nephrology

## Abstract

Several novel urinary kidney damage biomarkers predict the progression of kidney disease. However, the relations of these biomarkers to atherosclerosis, a major consequence of kidney disease, are less studied. Urinary levels of several biomarkers, including kidney injury molecule-1 (KIM-1), osteopontin, epidermal growth factor, and Dickkopf-3, were assessed in participants enrolled in the Swedish CArdioPulmonary BioImage Study. The study included 9,628 individuals with a mean age of 57.5 years, of which 52.4% were women. The presence of coronary artery stenosis and the coronary artery calcium score (CACS) were determined using coronary computed tomography angiography. To analyze the associations between coronary atherosclerosis and urinary biomarker levels, an ordered logistic regression model adusting for confounding factors was employed. KIM-1 was the only biomarker associated with both coronary stenosis and CACS after adjusting for established cardiovascular risk factors (odds ratio [95% confidence intervals], 1.23[1.05–1.44] and 1.25[1.07–1.47]). These results were consistent in sensitivity analyses of individuals without hypertension, diabetes, or known cardiovascular disease and with normal kidney function. Urinary KIM-1, a specific marker of proximal tubular damage, was robustly linked to coronary atherosclerosis even in apparently healthy individuals, which suggests that the detrimental interplay between the kidney and cardiovascular system begins before clinically overt kidney disease. Additional studies are warranted to evaluate the urinary KIM-1 to predict kidney and cardiovascular disease.

**Supplementary Information:**

The online version contains supplementary material available at 10.1038/s41598-024-80321-5.

## Introduction

Chronic kidney disease (CKD) significantly impacts global public health by directly causing morbidity and mortality, as well as being a major risk factor for cardiovascular disease (CVD)^1^. In fact, approximately half of the patients with advanced CKD have CVD, and cardiovascular mortality accounts for 40–50% of all deaths in patients with CKD^2^.

In clinical practice, creatinine-based estimated glomerular filtration rate (eGFR) and albuminuria are commonly used to diagnose and stage chronic kidney disease, but also for cardiovascular risk prediction^3^. However, both of these established biomarkers for kidney dysfunction and damage have limitations that should be taken into account when interpreting their clinical significance. Creatinine-based eGFR can be affected by factors such as age, muscle mass, and diet and could also be insensitive to nephron loss unless the injury is severe because of the temporal compensation effect of the single nephron glomerular filtration rate (SNGFR)^4,5^. Albuminuria, which mainly reflects glomerular damage, can be influenced by factors beyond kidney disease, such as diet, exercise, and stress, and may be a transient condition that changes over time^6^. Moreover, neither eGFR nor albuminuria directly reflects tubular damage or dysfunction, aspects of kidney pathology that may be important both for kidney disease progression and cardiovascular disease development^7^. Thus, a need for novel kidney biomarkers that may help improve CKD detection and prognostication has been proposed^7^.

In the present study, we highlight four promising novel kidney biomarkers under active investigation: urinary kidney injury molecule-1 (KIM-1), osteopontin, epidermal growth factor (EGF), and Dickkopf-3 (DKK-3). KIM-1 is a type 1 trans-membrane protein, whose expression is markedly up-regulated after renal proximal tubule injury^8^. Osteopontin (OPN) is a calcium binding protein which is expressed in bone, endothelial cells, and glomerular basement membrane^9^. In diabetic patients, plasma osteopontin level was significantly correlated with the degree of diabetic nephropathy^10^. Urinary EGF has recently been identified as a promising biomarker of chronic kidney disease (CKD) progression in adults with glomerular disease^11^. Low levels of EGF predict CKD progression and appear to reflect the extent of tubulointerstitial damage^11^. DKK-3 is a stress-induced, renal tubular epithelial cell-derived, pro-fibrotic molecule^12^. These previous findings highlighted the crucial role of these four novel biomarkers for reflecting timely kidney damage, but to what extent they are associated with atherosclerotic disease is less studied.

Therefore, we hypothesized that these four kidney biomarkers could reflect the burden of coronary atherosclerosis status due to their suggested interplay with renal and cardiovascular pathology. Accordingly, the present study aimed to investigate the association between urinary levels of KIM-1, osteopontin, DKK-3, EGF, and coronary artery atherosclerosis assessed by coronary computed tomography angiography (CCTA)^13^ and coronary artery calcium score (CACS)^14^. Second, we aimed to perform sensitivity analyses in individuals without known kidney disease, cardiovascular disease, hypertension or diabetes in order to investigate whether the detrimental interplay between the kidney and cardiovascular disease is already present before the overt disease. Third, we aimed to compare the predictive ability of these novel kidney biomarkers for coronary atherosclerosis to the established kidney biomarkers eGFR and albuminuria.

## Methods

### Study population

The participants were from the Uppsala site (*n* = 5,036) and Malmö site (*n* = 6,251) of the Swedish CArdioPulmonary BioImage Study (SCAPIS) cohort, a population-based study of cardiovascular and pulmonary diseases and their risk factors. In total, SCAPIS recruited 30,154 individuals aged 50–64 randomly from geographical areas surrounding 6 university hospitals across Sweden from 2013 to 2018. Each individual donated blood and urine for biobanking, questionnaires were administered and computed tomography imaging was performed. The biological samples were kept frozen at -80 degrees Celsius until analysis. The informed consent was obtained from all subjects and/or their legal guardian. The SCAPIS has been performed in accordance with the Declaration of Helsinki, and was approved by the ethical review board at Umeå University, Sweden (2010-228-31 M), and the ethical boards approved urine samples at Uppsala University and Lund University, Sweden (EPN Uppsala University 2016/387, and 2018/315; Lund University 2016/1031).

### Coronary atherosclerosis

The coronary atherosclerotic burden was measured with CCTA and CACS according to Agatston (Somatom Definition Flash, Siemens Medical Solutions, Solna, Sweden). Details regarding cardiac imaging have previously been described^15^.

The values of CCTA were defined as “No stenosis”, “Non-significant stenosis (< 50%)”, and “Significant stenosis ( > = 50%)”, by measuring the stenosis status of 18 segments of coronary arteries visually by experienced radiologists and cardiologists. Participants who had a stent in an artery or ever underwent CABG (Coronary Artery Bypass Graft) were defined as having significant stenosis > = 50%. If the calcification blooming was difficult to evaluate, it was defined as non-significant stenosis < 50%. Non-contrast enhanced images were applied to measure the total amount of calcifications in each artery and were summed to a total CACS according to international standards^16^. We divided the sum from CACS into five categories usually used in clinical practice (0, 0.1–100, 101–400, > 400). The detailed methods have been previously reported^26^.

### Assessment of urinary kidney damage biomarkers and covariates

At the first visit, venous blood and spot urine samples were collected after an 8-hour overnight fast. Spot urine samples were aliquoted and frozen at – 80 °C within two hours. Blood samples were immediately analyzed at the university hospital laboratory. Creatinine, glucose, and low-density lipoprotein cholesterol (LDL-C) was determined by direct measurement using Cobas 501 (Roche Diagnostics, Solna, Sweden). The urine sample was sent to the University Hospital of Uppsala for further analysis of the urinary kidney damage biomarkers. Urinary KIM-1 (DY1750B), Osteopontin (DY1433), DKK-3 (DY1118), and EGF (DY236) were analyzed by commercial sandwich ELISAs (R&D Systems, Minneapolis, MN, USA) according to the instructions of the manufacturer. Osteopontin and DKK-3 were analyzed in both cohorts, whereas KIM-1 and EGF were only analyzed in samples from the Uppsala site.

A value of urinary kidney damage biomarkers lower than the lower limit of quantification (LLQ) was imputed by LLQ/√2. The urinary biomarker to U-creatinine ratio was calculated for each biomarker (KIM-1, osteopontin, DKK, EGF, albumin) for adjusting urinary concentration. The CKD-EPI was used for calculating eGFR from serum creatinine, age and sex^17^.

### Other covariates

A validated and standardized questionnaire was used to collect sociodemographic, lifestyle, health, previous comorbidities, medical treatment for underlying diseases, and cardiovascular risk factors information in the SCAPIS population^26^. Recruitment center was also categorized as binary according to whether the participant was included in Malmö or Uppsala centers. Self-reported smoking status was categorized as never, former, and current smokers. Body mass index (BMI, kg/m^2^) was estimated by dividing weight (measured in kg) over the square of the height (measured in meters).

### Statistical analysis

Continuous baseline variables were presented as means (standard deviations) or median (interquartile range), while categorical variables were expressed as n (%). An ordered logistic regression analyzed the association between atherosclerosis and urinary biomarkers. Atherosclerosis levels were categorized into three groups in CCTA and four groups in CACS. To control for confounding, three models were used: model 1 adjusted for age, sex, country of birth, and individual cohort adjustments; model 2 further adjusted for eGFR and albuminuria; and model 3 adjusted for LDL-C, systolic and diastolic blood pressure, antihypertensive and anti-hyperlipidemia treatment, diabetes mellitus diagnosis, antidiabetic treatment, and smoking status. Sensitivity analyses were conducted using a generalized ordered logistic regression model to assess robustness and account for potential violations of assumptions. Forest plots illustrated the OR of atherosclerosis levels in both the main analysis and sensitivity analyses. Sensitivity analyses were performed for participants with specific criteria, including eGFR > 60 ml/min/1.73 m^2^, normoalbuminuria, self-reported non-hypertension, non-diabetes, and no known cardiovascular disease. Logistic regression assessed model performance using C-statistics and likelihood ratio test, comparing CCTA levels of ≥ 50% versus < 50% or no stenosis. We conducted further Spearman correlation analysis to explore the relationship between urinary biomarker levels, eGFR, and albuminuria. Statistical significance was set at a two-sided p-value < 0.05. All analyses utilized R (version 4.0.1) and Stata (version 15, College Station, TX, USA).

## Results

### Baseline characteristics

Among the 9,628 participants with available urinary kidney damage biomarker data in Uppsala (*n* = 4,717) and Malmö (*n* = 4,911), KIM-1 and EGF were only available in Uppsala, whereas osteopontin and DKK-3 were measured both in Uppsala and Malmö (Table 1). Participants enrolled had a mean age of 57.7 years in the Uppsala site, and 57.4 years in the Malmö site. The female population was 46.7% in the Malmö site and 48.7% in the Uppsala site. The CACS was higher in Malmö (71.5) than in Uppsala (53.5; Table 1). The number of participants with values below the lower limit of quantitation for each urinary biomarker was described as follows: U-KIM-1(*n* = 762), EGF(*n* = 10), U-osteopontin (*n* = 219), U-DKK-3 (*n* = 370), U-albumin (*n* = 2,368), and U-creatinine (*n* = 2) on Uppsala site; U-osteopontin (*n* = 209), U-DKK-3 (*n* = 734), U-albumin (*n* = 1,381), and U-creatinine (*n* = 15) on Malmö site. The correlations among the urinary biomarkers, eGFR and albuminuria were presented in Table S1.

**Table 1:** Baseline characteristics of participants categorized by enrollment site of the Swedish CArdioPulmonary bioImage study (SCAPIS) cohort.

	Enrollment site		
	Malmö (*N* = 4,911)	Uppsala (*N* = 4,717)	N
Age, years*	57.4 (4.3)	57.7 (4.4)	9,628
Female, n (%)	2,619 (53.3%)	2,422 (51.3%)	9,628
Country of birth, n (%)			9,590
Scandinavia	3,849 (78.7%)	4,233 (90.0%)	
Europe	670 (13.7%)	183 (3.9%)	
Asia	257 (5.3%)	189 (4.0%)	
Other area	112 (2.3%)	97 (2.1%)	
Smoking status, n(%)			9,628
Never smoker	2,089 (42.5%)	2,612 (55.4%)	
Former smoker	1,852 (37.7%)	1,445 (30.6%)	
Current smoker	846 (17.2%)	421 (8.9%)	
Diabetes Mellitus, n(%)	242 (5.1%)	197 (4.4%)	9,179
Body mass index*	27.3 (4.58)	27.0 (4.35)	9,628
Systolic blood pressure (mmHg)*	122 (16)	125 (16)	9,619
Diastolic blood pressure (mmHg)*	75 (10)	77 (10)	9,617
Plasma Glucose level (mg/dl)*	101 (23.3)	103 (18.2)	9,624
Albumin creatinine ratio (ug/mg)^†^	8.62 [3.32;21.2]	2.91 [1.12;10.7]	9,424
KIM-1/Cr (ng/mg)^†^	-	0.71 [0.33;1.28]	4,652
Osteopontin/Cr (pg/mg)^†^	828,852 [529,830;1172,904]	817,184 [526,880;1180,364]	9,541
DKK-3/Cr (pg/mg)^†^	415 [225;789]	475 [264;908]	9,541
EGF/Cr (pg/mg)^†^	-	20,290 [15,280;27,748]	4,654
Anti-hyperlipidemia treatment, n (%)	403 (8.6%)	348 (7.8%)	9,171
Anti-diabetes treatment, n (%)	206 (4.4%)	159 (3.6%)	9,169
CCTA:			9,187
Group 1 (no stenosis)	2,780 (58.2%)	2,638 (59.8%)	
Group 2 (< 50% stenosis)	1,653 (34.6%)	1,433 (32.5%)	
Group 3 ( > = 50% stenosis)	341 (7.1%)	342 (7.8%)	
Coronary artery calcium score (CACS) group, n (%)			9,272
Group 1 (= 0)	2,666 (56.1%)	2,806 (62.1%)	
Group 2 (0.1–100)	1,424 (30.0%)	1,216 (26.9%)	
Group 3 (101–400)	437 (9.2%)	337 (7.5%)	
Group 4 (> 401)	225 (4.7%)	161 (3.6%)	

CCTA: coronary computed tomography angiography.

CCTA group: 1: no stenosis/2: <50% stenosis or calcium blooming/3: >=50% stenosis or stent or CABG.

CACS group: Group 1 (CACS < 10)/ Group 2 (11–100)/ Group 3 (101–400)/ Group 4 (> 401).

Continuous variables are presented as *mean (standard deviation) or median [interquartile range] as appropriate. The median was presented for Albumin creatinine ratio, KIM-1/Cr, Osteopontin/Cr, DKK-3/Cr, and EGF/Cr.

## Kidney biomarkers and coronary stenosis (CCTA)

A total of 9,187 participants had complete CCTA data in Uppsala and Malmö. As seen in Table 2, a positive association was found between levels of urinary KIM-1 and more severe coronary stenosis after adjusting for age, sex, country of birth, and individual cohort (model 1) and after further adjustment for eGFR and albuminuria (Model 2) and also after further adjustment for low-density lipoprotein, systolic blood pressure, diastolic blood pressure, antihypertensive treatment, anti-hyperlipidemia treatment, diagnosis of diabetes mellitus, antidiabetic treatment, and smoke status (Model 3). Urinary osteopontin was also positively associated with coronary stenosis in models 1, 2, and 3 (Table 2). However, urinary EGF was inversely associated with coronary stenosis only in model 2, and DKK-3 was not associated with coronary stenosis in any model (Table 2).

**Table 2:** The association between kidney damage biomarkers and coronary computed tomography angiography scale group and coronary artery calcium score group in all participants.

Outcome	Model 1	Model 2	Model 3
	Odds ratio (95% CI)	Odds ratio (95% CI)	Odds ratio (95% CI)
	**CCTA group**		
KIM-1/Cr	**1.29 (1.11**,**1.50)**	**1.24 (1.07**,**1.45)**	**1.23 (1.05**,**1.44)**
Osteopontin/Cr	**1.11 (1.06**,**1.17)**	**1.10 (1.04**,**1.16)**	**1.07 (1.01**,**1.13)**
DKK-3/Cr	1.02 (0.98,1.07)	1.01 (0.96,1.05)	0.99 (0.95,1.03)
EGF/Cr	0.90 (0.79,1.03)	**0.82 (0.72**,**0.95)**	0.89 (0.77,1.03)
	**CACS group**		
KIM-1/Cr	**1.34 (1.16**,**1.56)**	**1.27 (1.09**,**1.47)**	**1.25 (1.07**,**1.47)**
Osteopontin/Cr	**1.10 (1.04**,**1.16)**	**1.07 (1.01**,**1.13)**	1.04 (0.98,1.09)
DKK-3/Cr	1.02 (0.97,1.06)	0.98 (0.94,1.02)	0.96 (0.92,1.00)
EGF/Cr	**0.83 (0.72**,**0.95)**	**0.77 (0.67**,**0.88)**	**0.84 (0.73**,**0.97)**

The odds ratio for CCTA/CACS group (95% CI) per 1-SD increment in kidney damage biomarker concentration.

CCTA group: 1: no stenosis/2: <50% stenosis or calcium blooming/3: >=50% stenosis or stent or CABG.

CACS group: Group 1 (CACS = 0)/ Group 2 (0.1–100)/ Group 3 (101–400)/ Group 4 (> 401).

Abbreviations: KIM-1: kidney injury molecule-1; DKK-3: Dickkopf-3; EGF: Epidermal growth factor, CI, confidence interval.

Model 1: Multivariable ordinal regression model with age, sex, country of birth, and individual cohort adjustments.

Model 2: Multivariable ordinal regression model adjusting for Model 1 covariates and additionally adjusting for eGFR and albuminuria.

Model 3: Multivariable ordinal regression model with adjusting for Model 2 covariates and additionally adjusting for low-density lipoprotein, systolic blood pressure, diastolic blood pressure, antihypertensive treatment, anti-hyperlipidemia treatment, diagnosis of diabetes mellitus, antidiabetic treatment, and smoke status.

### Kidney biomarkers and coronary artery calcium score (CACS)

A total of 9,272 participants had complete CACS data in Uppsala and Malmö. Similarly, a positive association was found between urinary biomarkers of urinary KIM-1 and CACS group in models 1, 2 and 3; an inverse association was found between EGF and CACS group in models 1, 2 and 3 (Table 2). However, urinary osteopontin was associated with the CACS group only in model 1 and model 2, and DKK-3 was not associated with the CACS group.

In summary, we observed a consistent positive association between KIM-1 and coronary artery stenosis defined by CCTA and CACS in all multivariable models. Urinary osteopontin was positively associated with CCTA but not CACS, whereas EGF was inversely associated with CACS but not CCTA in the fully adjusted model (Table 2).

### Sensitivity analyses

**Fig. 1:**
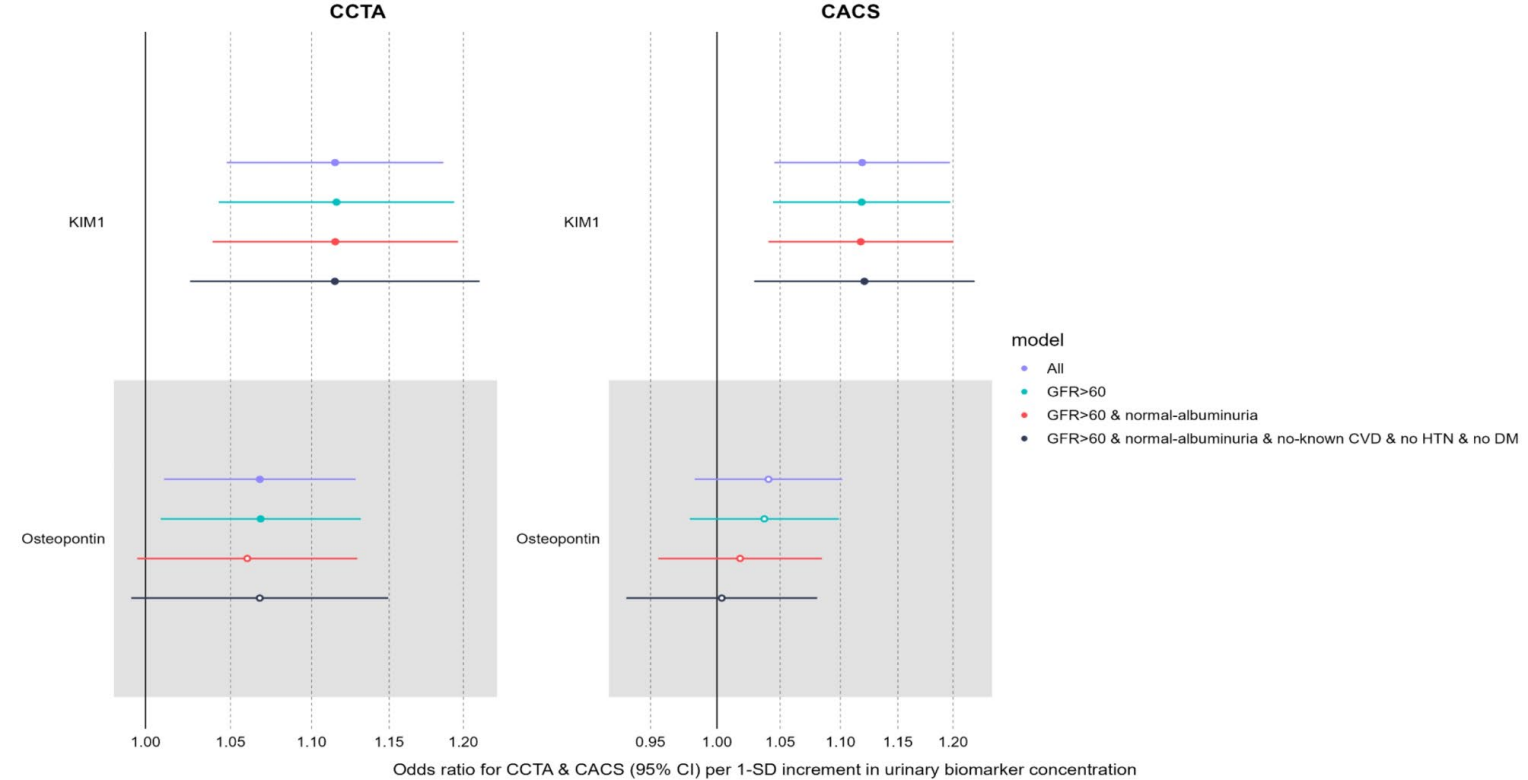
The forest plot of odds ratio for the association between kidney damage biomarkers and coronary stenosis defined by coronary computed tomography angiography (CCTA) and CACS. The analysis utilizes an ordinal regression model in main and subgroup analyses in the full adjustment model (Model 3). The odds ratio for CCTA (95% CI) per 1-SD increment in kidney damage biomarker concentration.

Three sensitivity analyses were performed to examine the association between urinary kidney damage biomarkers and coronary artery stenosis defined by CCTA and CACS. Figure 1 was the forest plot to visualize the comparison. We observed that the KIM-1 had a consistently positive association with CCTA and CACS in the subgroup population of participants with eGFR > 60 ml/min/1,73m^2^ (Table S2), further without albuminuria (Table S3), further without cardiovascular disease, hypertension or diabetes mellitus (Table S4) in the full adjustment model. Urinary osteopontin was positively associated with CCTA in the whole cohort (main analysis) and subgroup without kidney disease (Table S2) in the full adjustment model. Urinary DKK-3 was not associated with coronary artery stenosis in any sensitivity analysis. Urinary EGF was associated with CCTA in sensitivity analyses 1 and 3 and with CACS in the main analysis in the full adjustment model (Table S2, Table S4). A generalized ordered logistic regression did not alter the results.

### Model discrimination with biomarkers

In order to evaluate improvements in model discrimination, we separately added eGFR, albuminuria, KIM-1, osteopontin, EGF, and DKK-3 to a model with established cardiovascular risk factors (baseline model C-statistic = 0.747). As seen in Table 3, the addition of KIM-1 or eGFR improved the model fit compared to the baseline model but with minimal improvement in model discrimination (C-statistic = 0.748, likelihood ratio test *p* = 0.001 for both). Neither albuminuria nor any of the other kidney biomarkers improved model fit or discrimination (Table 3).

**Table 3:** Comparison of model performance for the prediction of coronary stenosis by adding kidney damage biomarkers to a baseline model including cardiovascular risk factors. The coronary stenosis was defined by coronary computed tomography angiography (CCTA).

Model	C-statistic (95%CI)	Likelihood ratio test *p*-value
Baseline model*	0.747 (0.734, 0.764)	Ref.
Baseline model + eGFR	0.748 (0.735, 0.765)	**< 0.001**
Baseline model + albuminuria	0.747 (0.734, 0.762)	0.486
Baseline model + KIM-1	0.748 (0.735, 0.763)	**0.001**
Baseline model + osteopontin	0.747 (0.733, 0.764)	0.124
Baseline model + EGF	0.747 (0.734, 0.763)	0.892
Baseline model + DKK-3	0.747 (0.734, 0.763)	0.882

*The baseline model was a multivariable logistic regression model with adjusting for age, sex, country of birth, and individual cohort, low-density lipoprotein, systolic blood pressure, diastolic blood pressure, antihypertensive treatment, anti-hyperlipidemia treatment, diagnosis of diabetes mellitus, antidiabetic treatment, and smoke status.

## Discussion

The current study is the first large population-based study to investigate the association of the kidney damage biomarkers KIM-1, DKK-3, osteopontin, and EGF with two indices of coronary artery atherosclerosis. The study demonstrated that urinary KIM-1 was robustly positively associated with both coronary stenosis and calcification as defined by CCTA and CACS, respectively. Interestingly, the association remained consistent even in apparently healthy individuals with normoalbuminuria and eGFR, without hypertension, diabetes or known CVD. In addition, urinary Osteopontin was positively associated with coronary stenosis defined by CCTA, whereas EGF was inversely associated with coronary calcification defined by CACS in the fully adjusted model. In contrast, DKK-3 was not associated with any indices of coronary atherosclerosis. As for the model prediction, the addition of KIM-1 to a model with established cardiovascular risk factors improved the model fit to a similar account as GFR, yet none of the established or experimental kidney biomarkers provided any meaningful increases in model discrimination as evaluated by the C-statistics analyses. These findings both confirm and extend our understanding of the importance of the kidney for the development of cardiovascular disease and suggest that there may be important aspects of kidney pathology that are not reflected by the established kidney biomarkers albuminuria and eGFR.

There are a few previous studies that have reported associations between the present novel kidney biomarkers and cardiovascular phenotypes: Plasma DKK-3 has been reported to be inversely associated with atherosclerotic calcified plaque and coronary atherosclerosis in previous small-scale studies and considered as an independent factor in predicting the presence of coronary atherosclerosis in the general population and patients with type 2 diabetes^18^. Plasma osteopontin was recognized as an indicator for early coronary vascular calcification in diabetic kidney disease (DKD)^19^. Plasma osteopontin also predicts cardiovascular death^20^ and major cardiovascular disease^21^. Endogenous osteopontin could prevent phosphate-induced nephrocalcinosis and vascular calcification in patients with CKD^22^, but urinary osteopontin was not associated with CVD mortality in elderly men^20^.

In the Chronic Renal Insufficiency Cohort (CRIC) Study, researchers identified urinary KIM-1 as a biomarker independently associated with atherosclerotic cardiovascular events and all-cause death in CKD patients^23^. In another study in elderly men from the general population, urinary KIM-1 predicted cardiovascular mortality independently of and additive to eGFR and albuminuria^24^.

However, in a multi-ethnic study, urinary KIM-1 was not associated with subclinical measures of cardiovascular disease in individuals free of cardiovascular disease at baseline^25^.

Kidney injury molecule-1 is a type 1 membrane protein most predominantly present in the proximal tubule of the injured kidney^26^. KIM-1 was secreted from proximal renal tubular epithelial cells during the kidney injury caused by ischemia, hypoxia and toxicity, and its level refers to the extent of kidney damage^27^. Studies in animals have shown that KIM-1 is produced in response to ischemia that may reflect kidney response to a systemic influence or a systemic response to ongoing proximal tubule injury^28^. In the very beginning of the acute kidney injury, KIM-1 is released by tubular cells and mediates phagocytosis, helps repair the tubular injury^29^, and regulates inflammatory response. KIM-1 is involved in renal repair by activating the ERK/ MAPK signaling pathway, suppressing Gα12 activation, and blocking GTP loading^29^. On the contrary, instead of protecting effect, persistent KIM-1 secretion in chronic kidney disease could promote renal fibrosis by promoting the secretion of monocyte chemotactic protein 1 (MCP-1)^30^. In addition, the KIM-1 gene can also be expressed in the immune cell^31^. It has also been reported to be linked with immune disorders, such as asthma, ectopic dermatitis, rheumatoid arthritis, and systemic lupus erythematosus^32,33^. KIM-1 was found to be associated with IL10 expression to regulate TH2 immune response in patients with SLE^34^ or allergic rhinitis^35^. It was also reported to increase tumor necrosis factor-alpha (TNFα) and IL6^36^ by stimulating macrophages, which indicates its essential role in inflammatory and autoimmune regulation. In the context of CVD, In the context of CVD, KIM-1 has been linked to an increased risk of chronic heart failure in community-healthy adults^37^. Additionally, higher urinary KIM-1/creatinine has been associated with a higher risk of cardiovascular mortality^24^. However, the relationship between KIM-1 and cardiovascular disease is complex and may involve multiple pathways.To summarize, although urinary KIM-1 is predominantly suggested to reflect proximal tubular damage, previous studies show that KIM-1 is involved in various pathophysiological mechanisms in renal, cardiovascular and autoimmune diseases. The regulation of inflammation and immunity could be involved in the process of atherosclerosis formation. Further studies to investigate the expression of KIM-1 in the process of atherosclerosis and the role of tubular damage and dysfunction should be done in the future.

Patients with CKD have a markedly increased risk for cardiovascular events^38^, and even in the early stages of chronic kidney disease, the risk of cardiovascular events caused by renal disease grows dramatically^39^. Since coronary artery atherosclerosis has been recognized as an independent predictor of cardiovascular disease and mortality^40^, early detections of coronary atherosclerosis could raise the cognition of potential cardiovascular risk, encouraging people to keep healthy lifestyle modifications, therefore decreasing the incidence of cardiovascular morbidity and mortality^41^. Our findings suggest that incorporating urinary KIM-1 in a model that already includes established cardiovascular risk factors enhances the model fit to a degree comparable to that of GFR. However, additional studies with a larger heterogeneity regarding kidney disease and cardiovascular disease are needed to fully evaluate whether there are any clinical implications of using urinary KIM-1 for risk prediction purposes for sub-clinical atherosclerosis or future cardiovascular events. Additionally, we found an association between urinary KIM-1 and coronary atherosclerosis in apparently healthy individuals. Taken together, these findings suggest that there is room for improvement, both in the prediction of cardiovascular complications but also in the detection of early kidney disease beyond eGFR and albuminuria. Our findings put forward urinary KIM-1 as the potential promising biomarker candidate of the four biomarkers evaluated in the present study.

Major strengths include the large-scale population study with state-of-art measurements of coronary atherosclerosis, multiple kidney damage biomarkers, and the detailed characterization of the study participants regarding lifestyle and risk factors. There are also several limitations that need to be taken into account in the interpretation of our findings. First, the cross-sectional study design precludes causal inference of our findings. Second, no validation analysis was performed in the present study. Because the urinary KIM-1 and osteopontin were only measured at the Uppsala site, we did not perform a discovery-validation study design. Yet our study cohort is by far the largest to date evaluating the association between urinary kidney biomarkers and coronary atherosclerosis.

In conclusion, Urinary KIM-1, a specific marker of proximal tubular damage, was robustly linked to coronary atherosclerosis even in apparently healthy individuals, suggesting that the detrimental interplay between the kidney and cardiovascular system begins before clinically overt kidney disease. Additional studies are warranted to evaluate the clinical utility of urinary KIM-1 and other novel kidney damage biomarkers for diagnostic and risk prediction purposes.

## Electronic supplementary material

Below is the link to the electronic supplementary material.

Supplementary Material 1

## Data Availability

The personal data in SCAPIS are of sensitive nature and therefore cannot be made freely available. However, by contacting the corresponding author or study organization (www.scapis.org), sharing of data can be arranged for reproducing study results and procedures.

## References

[1] GBDCKD Collaboration. Global, regional, and national burden of chronic kidney disease, 1990–2017: A systematic analysis for the global burden of disease study 2017. *Lancet* **395**, 709–733. 10.1016/S0140-6736(20)30045-3 (2020). 32061315 10.1016/S0140-6736(20)30045-3 PMC7049905

[2] Webster, A. C., Nagler, E. V., Morton, R. L. & Masson, P. Chronic kidney disease. *Lancet* **389**, 1238–1252. 10.1016/S0140-6736(16)32064-5 (2017). 10.1016/S0140-6736(16)32064-5 27887750

[3] Pasternak, M. et al. Association of albuminuria and regression of chronic kidney disease in adults with newly diagnosed moderate to severe chronic kidney disease. *JAMA Netw. Open.* **5**, e2225821. 10.1001/jamanetworkopen.2022.25821 (2022). 35943741 10.1001/jamanetworkopen.2022.25821 PMC9364131

[4] Wright, F. S. & Giebisch, G. Glomerular filtration in single nephrons. *Kidney Int.* **1**, 201–209. 10.1038/ki.1972.30 (1972). 4671212 10.1038/ki.1972.30

[5] Nankivell, B. J., Nankivell, L. F. J., Elder, G. J. & Gruenewald, S. M. How unmeasured muscle mass affects estimated GFR and diagnostic inaccuracy. *EClinicalMedicine* **29-30**, 100662. 10.1016/j.eclinm.2020.100662 (2020). 33437955 10.1016/j.eclinm.2020.100662 PMC7788434

[6] Waikar, S. S. et al. Biological variability of estimated GFR and albuminuria in CKD. *Am. J. Kidney Dis.* **72**, 538–546. 10.1053/j.ajkd.2018.04.023 (2018). 30031564 10.1053/j.ajkd.2018.04.023 PMC6469385

[7] Zhang, W. R. & Parikh, C. R. Biomarkers of acute and chronic kidney disease. *Annu. Rev. Physiol.* **81**, 309–333. 10.1146/annurev-physiol-020518-114605 (2019). 30742783 10.1146/annurev-physiol-020518-114605 PMC7879424

[8] Han, W. K., Bailly, V., Abichandani, R., Thadhani, R. & Bonventre, J. V. Kidney injury molecule-1 (KIM-1): A novel biomarker for human renal proximal tubule injury. *Kidney Int.* **62**, 237–244. 10.1046/j.1523-1755.2002.00433.x (2002). 12081583 10.1046/j.1523-1755.2002.00433.x

[9] Fitzpatrick, L. A., Severson, A., Edwards, W. D. & Ingram, R. T. Diffuse calcification in human coronary arteries. Association of osteopontin with atherosclerosis. *J. Clin. Invest.* **94**, 1597–1604. 10.1172/JCI117501 (1994). 7929835 10.1172/JCI117501 PMC295319

[10] Zhou, C. et al. Blockade of osteopontin inhibits glomerular fibrosis in a model of anti-glomerular basement membrane glomerulonephritis. *Am. J. Nephrol.* **32**, 324–331. 10.1159/000319490 (2010). 20720406 10.1159/000319490 PMC2969149

[11] Azukaitis, K. et al. Low levels of urinary epidermal growth factor predict chronic kidney disease progression in children. *Kidney Int.* **96**, 214–221. 10.1016/j.kint.2019.01.035 (2019). 31005273 10.1016/j.kint.2019.01.035

[12] Schunk, S. J., Speer, T., Petrakis, I. & Fliser, D. Dickkopf 3-a novel biomarker of the ‘kidney injury continuum’. *Nephrol. Dial Transplant.* **36**, 761–767. 10.1093/ndt/gfaa003 (2021). 32025732 10.1093/ndt/gfaa003

[13] Patel, M. R. et al. Prevalence and predictors of nonobstructive coronary artery disease identified with coronary angiography in contemporary clinical practice. *Am Heart J* **167**, 846–852 e842 10.1016/j.ahj.2014.03.001 (2014). 10.1016/j.ahj.2014.03.001 24890534

[14] Greenland, P., Blaha, M. J., Budoff, M. J., Erbel, R. & Watson, K. E. Coronary calcium score and cardiovascular risk. *J. Am. Coll. Cardiol.* **72**, 434–447. 10.1016/j.jacc.2018.05.027 (2018). 30025580 10.1016/j.jacc.2018.05.027 PMC6056023

[15] Bergstrom, G. et al. Prevalence of subclinical coronary artery atherosclerosis in the general population. *Circulation* **144**, 916–929. 10.1161/CIRCULATIONAHA.121.055340 (2021). 34543072 10.1161/CIRCULATIONAHA.121.055340 PMC8448414

[16] Agatston, A. S. et al. Quantification of coronary artery calcium using ultrafast computed tomography. *J. Am. Coll. Cardiol.* **15**, 827–832. 10.1016/0735-1097(90)90282-t (1990). 2407762 10.1016/0735-1097(90)90282-t

[17] Inker, L. A. et al. New creatinine- and cystatin C-based equations to estimate GFR without race. *N Engl. J. Med.* **385**, 1737–1749. 10.1056/NEJMoa2102953 (2021). 34554658 10.1056/NEJMoa2102953 PMC8822996

[18] Register, T. C. et al. Plasma Dickkopf1 (DKK1) concentrations negatively associate with atherosclerotic calcified plaque in African-Americans with type 2 diabetes. *J. Clin. Endocrinol. Metab.* **98**, E60–65. 10.1210/jc.2012-3038 (2013). 23125289 10.1210/jc.2012-3038 PMC3537092

[19] Berezin, A. E. & Kremzer, A. A. Circulating osteopontin as a marker of early coronary vascular calcification in type two diabetes mellitus patients with known asymptomatic coronary artery disease. *Atherosclerosis* **229**, 475–481. 10.1016/j.atherosclerosis.2013.06.003 (2013). 23880208 10.1016/j.atherosclerosis.2013.06.003

[20] Feldreich, T. et al. Urinary osteopontin predicts incident chronic kidney disease, while plasma osteopontin predicts cardiovascular death in elderly men. *Cardiorenal Med.* **7**, 245–254. 10.1159/000476001 (2017). 28736565 10.1159/000476001 PMC5511996

[21] Sponder, M. et al. Osteopontin is elevated in patients with mitral annulus calcification independent from classic cardiovascular risk factors. *BMC Cardiovasc. Disord*. **16**, 132. 10.1186/s12872-016-0314-3 (2016). 27283399 10.1186/s12872-016-0314-3 PMC4901469

[22] Paloian, N. J., Leaf, E. M. & Giachelli, C. M. Osteopontin protects against high phosphate-induced nephrocalcinosis and vascular calcification. *Kidney Int.* **89**, 1027–1036. 10.1016/j.kint.2015.12.046 (2016). 27083280 10.1016/j.kint.2015.12.046 PMC4834144

[23] Park, M. et al. Urine kidney injury biomarkers and risks of cardiovascular disease events and all-cause death: The CRIC study. *Clin. J. Am. Soc. Nephrol.* **12**, 761–771. 10.2215/CJN.08560816 (2017). 28254771 10.2215/CJN.08560816 PMC5477212

[24] Carlsson, A. C. et al. Urinary kidney injury molecule-1 and the risk of cardiovascular mortality in elderly men. *Clin. J. Am. Soc. Nephrol.* **9**, 1393–1401. 10.2215/CJN.11901113 (2014). 24923577 10.2215/CJN.11901113 PMC4123404

[25] Park, M. et al. Associations of kidney injury markers with subclinical cardiovascular disease: The multi-ethnic study of atherosclerosis. *Clin. Nephrol.* **84**, 358–363. 10.5414/CN108668 (2015). 26558369 10.5414/CN108668 PMC4776253

[26] Bonventre, J. V. & Yang, L. Kidney injury molecule-1. *Curr. Opin. Crit. Care*. **16**, 556–561. 10.1097/MCC.0b013e32834008d3 (2010). 20930626 10.1097/MCC.0b013e32834008d3

[27] Bonventre, J. V. Kidney injury molecule-1 (KIM-1): A urinary biomarker and much more. *Nephrol. Dial Transpl.* **24**, 3265–3268. 10.1093/ndt/gfp010 (2009). 10.1093/ndt/gfp010 19318357

[28] Ko, G. J. et al. Transcriptional analysis of kidneys during repair from AKI reveals possible roles for NGAL and KIM-1 as biomarkers of AKI-to-CKD transition. *Am. J. Physiol. Ren. Physiol.* **298**, F1472–1483. 10.1152/ajprenal.00619.2009 (2010). 10.1152/ajprenal.00619.2009 20181666

[29] Zhang, Z. & Cai, C. X. Kidney injury molecule-1 (KIM-1) mediates renal epithelial cell repair via ERK MAPK signaling pathway. *Mol. Cell. Biochem.* **416**, 109–116. 10.1007/s11010-016-2700-7 (2016). 27084535 10.1007/s11010-016-2700-7 PMC4883006

[30] Humphreys, B. D. et al. Chronic epithelial kidney injury molecule-1 expression causes murine kidney fibrosis. *J. Clin. Invest.* **123**, 4023–4035. 10.1172/JCI45361 (2013). 23979159 10.1172/JCI45361 PMC3755983

[31] Xiao, S. et al. Tim-1 stimulation of dendritic cells regulates the balance between effector and regulatory T cells. *Eur. J. Immunol.* **41**, 1539–1549. 10.1002/eji.201040993 (2011). 21469101 10.1002/eji.201040993 PMC3129006

[32] Kim, H. Y. et al. T-cell immunoglobulin and mucin domain 1 deficiency eliminates airway hyperreactivity triggered by the recognition of airway cell death. *J. Allergy Clin. Immunol.* **132** (e416), 414–425. 10.1016/j.jaci.2013.03.025 (2013). 23672783 10.1016/j.jaci.2013.03.025 PMC3732546

[33] Wang, Y. et al. Expression of human TIM-1 and TIM-3 on lymphocytes from systemic lupus erythematosus patients. *Scand. J. Immunol.* **67**, 63–70. 10.1111/j.1365-3083.2007.02038.x (2008). 18052965 10.1111/j.1365-3083.2007.02038.x

[34] Nakae, S. et al. TIM-1 and TIM-3 enhancement of Th2 cytokine production by mast cells. *Blood* **110**, 2565–2568. 10.1182/blood-2006-11-058800 (2007). 17620455 10.1182/blood-2006-11-058800 PMC1988955

[35] Xu, G. et al. Expression of T-cell immunoglobulin- and mucin-domain-containing molecule-1 (TIM-1) is increased in a mouse model of asthma and relationship to GATA-3. *Life Sci.* **82**, 663–669. 10.1016/j.lfs.2007.12.017 (2008). 18234236 10.1016/j.lfs.2007.12.017

[36] Hein, R. M. & Woods, M. L. TIM-1 regulates macrophage cytokine production and B7 family member expression. *Immunol. Lett.* **108**, 103–108. 10.1016/j.imlet.2006.11.004 (2007). 17161870 10.1016/j.imlet.2006.11.004

[37] Carlsson, A. C. et al. Urinary kidney injury molecule 1 and incidence of heart failure in elderly men. *Eur. J. Heart Fail.* **15**, 441–446. 10.1093/eurjhf/hfs187 (2013). 23220287 10.1093/eurjhf/hfs187

[38] Stevens, P. E. et al. Chronic kidney disease management in the United Kingdom: NEOERICA project results. *Kidney Int.* **72**, 92–99. 10.1038/sj.ki.5002273 (2007). 17440495 10.1038/sj.ki.5002273

[39] Valdivielso, J. M. et al. Atherosclerosis in chronic kidney disease: More, less, or just different? *Arterioscler. Thromb. Vasc Biol.* **39**, 1938–1966. 10.1161/ATVBAHA.119.312705 (2019). 31412740 10.1161/ATVBAHA.119.312705

[40] Barrett-Connor, E. L. Obesity, atherosclerosis, and coronary artery disease. *Ann. Intern. Med.* **103**, 1010–1019. 10.7326/0003-4819-103-6-1010 (1985). 3904565 10.7326/0003-4819-103-6-1010

[41] Khan, M. A. et al. Global epidemiology of ischemic heart disease: Results from the global burden of disease study. *Cureus* **12**, e9349. 10.7759/cureus.9349 (2020). 32742886 10.7759/cureus.9349 PMC7384703

